# Evaluation of an on-site surface enhanced Raman scattering sensor for benzotriazole

**DOI:** 10.1038/s41598-020-65181-z

**Published:** 2020-05-19

**Authors:** Florian Wieduwilt, Christoph Lenth, Georgios Ctistis, Ulrich Plachetka, Michael Möller, Hainer Wackerbarth

**Affiliations:** 1 Laser-Laboratorium Göttingen e.V., Hans-Adolf-Krebs-Weg 1, 37077 Göttingen, Germany; 2 AMO GmbH, Otto-Blumenthal-Straße 25, 52074 Aachen, Germany

**Keywords:** Analytical chemistry, Environmental chemistry, Materials for optics, Structural materials, Nanoscale materials, Techniques and instrumentation, Applied optics, Optical materials and structures, Optical techniques, Other photonics

## Abstract

Benzotriazole (BTAH) has been used for decades as corrosion inhibitor and antifreeze. Since it is fairly soluble in water but very stable and can only be partly removed from wastewater treatment plants, it represents a threat to the environment and thus also to human health. Therefore, it is of uttermost importance to have a detection method capable of monitoring the concentration of BTAH at trace level on-site. Here, we demonstrate that a sensor based on surface-enhanced Raman spectroscopy is capable of detecting trace-level concentrations of BTAH. We carefully studied the concentration dependency and the time dependent coverage. Moreover, we could not only perform the measurements with clean solution but also with real samples from a wastewater treatment plant, ensuring that our method proposed works in a complex environment.

## Introduction

Benzotriazole (BTAH) is a versatile chemical compound. It has been known for seventy years as an effective corrosion inhibitor for copper and its alloys by preventing undesirable surface reactions^1^. Moreover, BTAH is used in dishwashing detergents as silver protection, as well as in anti-freeze, heating and cooling systems, hydraulic fluids and also in vapor phase inhibitors^2^, leading to a production of $1000\mbox{--}10000$ t per year (only) in Europe. The most noteworthy characteristics are however, that BTAH is fairly soluble in water, not readily degradable and has a limited sorption tendency. Therefore, and due to the large variety of applications where BTAH is used, it is found ubiquitously in aquatic systems and can only be partly removed in wastewater treatment plants. In the greater Beijing area with 20 million people, for example, $1.0\,\mu g/l$ were found in wastewater^3^. Furthermore, BTAH has been found in lakes and rivers in Switzerland in concentrations ranging between $0.1\,\mu g/l$ and $6.3\,\mu g/l$ respectively^4^. Hence it follows that, as an identified micro-pollutant, it is of a growing concern in the water resources being threatened in their biodiversity as well as human health^5–7^. This is why an early detection of BTAH at trace-level concentration can critically avail the environmental monitoring.

In order to detect BTAH, sophisticated mass spectroscopy is established at present. Since on-site analysis is becoming more and more important, another appropriate method is urgently needed. Surface-enhanced Raman scattering (SERS) is predestined for this issue, since it is a sensitive spectroscopic method enabling the detection of molecular analytes down to the attogram level^8^. This is particularly attractive because it combines high sensitivity with high information content for establishing molecular identity.

The essential plasmonic nanostructures are generated by various procedures including the fabrication of plasmonic nanoparticle arrays assembled by a seed-mediated electroless plating method or single pulse UV-Laser treatment^9,10^. Fan *et al*. described SERS platforms using nanolithography methods in an overview, including electron-beam (e-beam) lithography and focused ion beam (FIB) as well as template-based methodologies to generate metallic nano-patterns^11^. Mosier-Boss reviewed the fabrication of the most common SERS-active substrates used. Three generic categories are classified: (1) metal nanoparticles in suspension; (2) metal nanoparticles immobilized on solid substrates; and (3) nanostructures fabricated directly on solid substrates by nanolithography and template based synthesis^12^.

The potential of SERS as an analytical application has been intensively explored during the last three decades using different kind of substrates or nanoparticles^13^. Examples are the explosive detection for security applications and analyte detection in medical applications^13–15^. This large interest has led to a commercialization of the technique leading to robust portable Raman spectrometers as well as commercially available SERS-substrates for on-site analysis. One of the first commercially available SERS-substrates was based on conventional optical lithography followed by anisotropic etching of a silicon wafer to generate inverted pyramidal pits finally covered by a thin gold layer^16^. Schmidt and coworkers fabricated a large area of leaning nanopillars by a maskless reactive ion etch process, providing a high aspect ratio. The coating with silver is performed by electron beam evaporation^17^. This substrate is applied for the analysis of volatile organic compounds and hydrogen cyanide in human breath^18,19^.

However, to our knowledge, there is not any analytical application running based on SERS. One argument hampering the versatile application is that the analyte must bind to the hot-spots of the SERS-substrates. Rhodamine and thiols often used in SERS studies have a strong surface binding affinity. However, many practical relevant analytes only bind weakly to the surface, leading to low signal-to-noise ratios.

Here, we show our experiments with BTAH as an analyte, which, through its strong surface binding affinity, is predestined for SERS^20^. We thereby evaluate SERS-substrates for their usability for on-site analysis.

## Materials and Methods

### Materials

**Figure 1:**
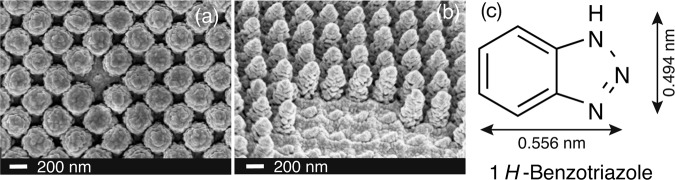
(**a**) Top view scanning electron micrograph of the nanostructured sample AMO C7. The pillar diameter is 200 nm and the pillar height 337 nm, respectively. (**b**) Tilted angle view of (a). It is visible that the pillars are cone shaped. (**c**) Chemical structure of BTAH. In addition length scales are added, which are used in the calculation for the amount being detected in the laser-spot area.

Benzotriazole (BTAH) with purity ReagentPlus ($99\, \%$) was purchased from Sigma Aldrich Inc. Benzotriazole consist of a benzene ring and, most importantly, has three nitrogen atoms. The molecule shows an affinity to noble metals, due to the free electron pairs (at the nitrogen atoms), which can undergo a dative bond, see Fig. 1(c). Concentration measurements were performed by dissolving BTAH in ultrapure water (Milli-Q, Millipore Corporation). The real water samples were taken from a wastewater treatment plant in Ochtrup, Germany on Aug. 15, 2018 at the drainage after a flocculation filtration step. They have a BTAH concentration of $8.80\,\mu g/l$.

### SERS-Substrates

The main SERS-substrates of this study (AMO C7) were prepared on silicon dioxide. In short, the SERS-active structures were prepared by soft-UV-nano-imprinting into Amonil resist on glass wafers^21,22^. The structures were then etched 700 nm into the SiO_2_-substrate using reactive ion-etching (RIE) with a CHF_3_-plasma. In this process the resist mask is virtually used up during the etching procedure, thus cleaning processes are obsolete. At the end, a gold layer of 200 nm was evaporated on top with a rate of 1.1Å/s at *p* = 1.87 × 10^−5 ^*mbar* using physical vapor deposition (Auto 306, BOC Edwards, UK). The resulting conical nanopillar structures of the so-called C7 substrates are shown in SEM-images in Fig. 1, where panel (a) shows the pillars in top view and (b) under a tilt angle of ${15}^{\circ }$. The samples have a rectangular pattern of the pillars with a pitch size of 375 nm. The pillar diameter is approximately 200 nm and their height is 337 nm, respectively. The active area of a single substrate is $7\times 11\,m{m}^{2}$.

Furthermore, we investigated the applicability of two commercially acquired SERS-substrates for the BTAH-detection: First, the substrates from AtoID (http://www.atoid.com, Lithuania) were used. They exist in two different versions, called “MatoS” (gold coating) and “RandaS” (silver coating). The active SERS area of $5\times 3\,m{m}^{2}$ was fabricated using ultra-short pulse laser ablation directly on the silver- and gold-coated soda-lime glass substrate, respectively. The resulting structure is stochastically nano-patterned with features between a few nm up to a micron in size^23^. Second, the “SERStrate” substrates from Silmeco (https://www.silmeco.com, Denmark), are made of silicon nanopillars coated with either gold or silver (vide supra). They exhibit an active area of $4\times 4\,m{m}^{2}$. A two step process is used to make these substrates. First, mask-less dry-etching is done to create the silicon nanopillars followed by electron beam evaporation of gold or silver to coat the silicon^24^.

### Instrumentation

**Figure 2:**
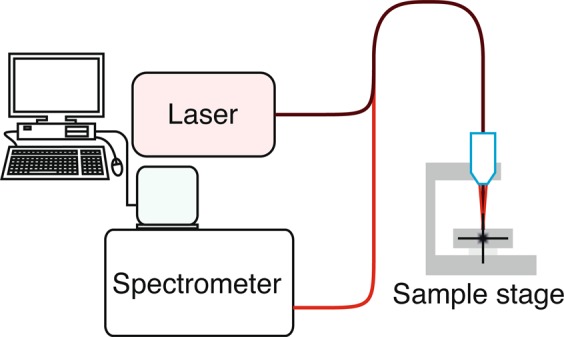
Schematic of the experimental setup. The sample stage leaves the opportunity to move the sample in x,y, and z direction for scanning purposes.

The Raman measurements were performed with a standard Raman system (Kaiser Optical Systems Inc., Ann Arbor, MI, USA) with an excitation wavelength of $\lambda \mathrm{=785}\,nm$. The incident power of the laser emission was set to 12.5 mW at the probe head. Figure 2 shows thereby schematically the experimental setup. A second measurement run was performed using a portable Raman spectrometer i-RamanPro (B&W Tek, Inc. Model: BWS475–785S) with an operating wavelength of $\lambda \mathrm{=785}\,nm$ and a laser power of $12.5\,mW$ at the probe head. Analysis was performed using ORIGIN Pro software.

### Measurement routine

To ensure that BTAH is uniformly distributed over the active area of the substrates and to account for inhomogeneities of the substrate, a measurement routine involving point measurements on five different positions on the substrate was used. To achieve this, the substrate was positioned on a x,y,z-positioning stage with sub-$\mu$ m accuracy. The distance to the excitation (z-axis) was regulated on maximum signal intensity of the bare substrate. Each spectrum was taken with $10\,$ s acquisition time and three accumulations to enhance signal-to-noise ratio. The acquired spectra of all five measurement points were then averaged to a single spectrum. For the concentration dependent measurements, the substrates were laid in $0.8\,ml$ of BTAH solution with a specific concentration for 15 min. After taking the sample out, we waited until the residual liquid evaporated (approximately 5 min) before starting the Raman measurements. The time-resolved measurements were performed on a single point. The acquisition time was reduced to $3\,s$ with two accumulations leading to a total measurement time of $30\,s$ for each time step. For this measurement, the substrate was placed in a glass beaker with 5 ml Milli-Q water. Then $0.8\,ml$ BTAH were added (concentration $10\,mg/l$) to the water and a spectrum was taken every $30\,{\rm{s}}$. At the end, a negative measurement was performed using a flat gold mirror.

For real wastewater samples, a concentration procedure was deemed necessary. For this, $20\,ml$ of the sample were evaporated to $10\,ml$ ($5\,ml$) achieving a doubling (quadruplicating) of the concentration. The measurements were then performed on the residue.

## Results and Discussion

### Concentration dependent measurements

First, we performed a concentration dependent measurement series to determine the detection limit of the BTAH on the SERS-active substrates. For this we used our C7 substrates, which are described in the SERS-Substrates section. The measurement routine is as described in the experimental section.

**Figure 3:**
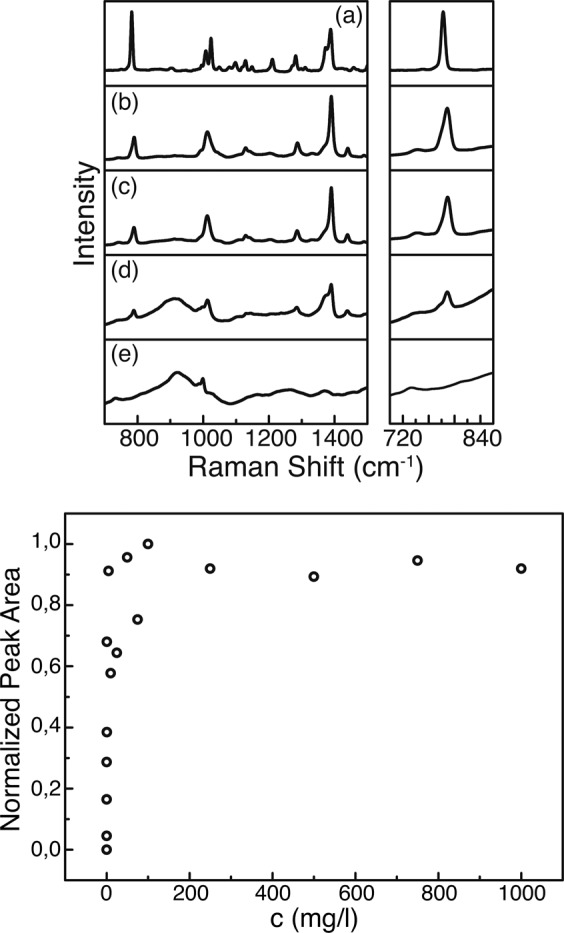
Top: Raman spectra for different concentrations of BTAH measured with the Kaiser system. At the right, the area around $780\,c{m}^{-1}$ is magnified, showing the evolution of the benzene breathing mode. (**a**) Pure BTAH as reference. (**b**–**d**) Decreasing concentration of BTAH from 1000 over 10 to $0.1\,mg/l$, respectively. (**e**) Pure C7 substrate. Bottom: Normalized peak area of the $783\,c{m}^{-1}$ peak versus concentration of BTAH revealing the limit of detection.

Figure 3 shows the experimental results for this substrate. The concentration series starts from the pure BTAH signal (a) reducing to the bare substrate (e), the latter giving the background signal for a better analysis. The spectra are shown in Fig. 3 (top). It is apparent that not all vibrational modes of BTAH visible in the pure sample can be taken for identification purposes, because of an overlap with bands of the substrate. Therefore, the analysis concentrates on the bands with a strong response at $783\,c{m}^{-1}$ and at $1387\,c{m}^{-1}$, representing the benzene breathing mode and the triazole ring stretching mode, respectively^21^. Thereby, the benzene breathing mode has been used as reference peak for the determination of the detection limit. Decreasing the concentration, the peak height as well as its integrated area are decreasing. The latter value has been used to quantify the detectability, as it is more robust against noise or thermal drifts of the center frequency of the mode.

The peak is thereby fitted with a Lorentzian profile after a background correction (ALS, asymmetric least squares), assuming no coupling between adjacent vibrational modes or between a mode and the background continuum, the latter justified because no Fano-like shape of the peak can be observed. The result of the analysis is shown in Fig. 3 (bottom). It is visible that an increase of the BTAH concentration above $100\,mg/l$ does not lead to a further increase in peak area. In fact, there is a saturation behavior indicating that the BTAH layer is thick enough to mask any signal originating from the substrate. The detection limit determined with this method lies considerably beneath $0.10\,mg/l$. The error margin of the analyzed concentrations lies in between $1\, \%$ and $2\, \%$, which would result in error bars smaller than the symbols of the data points and therefore are not visible in Fig. 3 (bottom). With these results, the measurement routine was directly transferred to an examination of real wastewater samples.

### Time-resolved measurements

After determining the detection limit for the pure BTAH in the previous section, the question still to be answered is how the BTAH is adsorbing on the SERS-substrates and how this process could be possibly described, i.e. Langmuir or Freundlich isothermal behavior. This is possible because SERS can detect the surface coverage at ultralow concentrations^20^.

**Figure 4:**
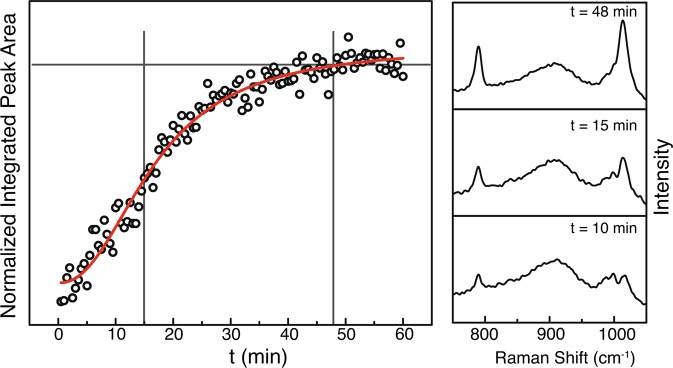
Time resolved adsorption behavior of BATH onto the SERS-substrate at room temperature. At each time a spectrum was taken (spectra on the right) and the peak area of the benzene breathing mode at $783\,c{m}^{-1}$ is plotted versus time. In red, a fit using a Hill-Langmuir function, is performed. Right: Spectra at three different times. At $t\,\mathrm{=\; 48}$ min the saturation of the peak area starts, at $t\,\mathrm{=\; 15}$ min the BTAH can be unambiguously detected. At $t\,\mathrm{=\; 10}$ min the BTAH peak is visible but to small to be used as reference.

We, therefore, designed our experiment such that we could monitor the adsorption over a long period of time (60 min). The acquired spectra were again analyzed by fitting the width of the benzene breathing mode at $783\,c{m}^{-1}$. Figure 4 shows the resulting graph. One can see a monotonic increase of the peak intensity up to 48 min. Then the intensity equals a plateau indicating a saturated surface coverage masking again signals resulting from the SERS-substrates.

From the experiment another critical time can be deduced. It is the minimal time necessary to be able to detect the BTAH. As shown exemplarily in the spectra of Fig. 4, the minimum exposure time lies at around 15 min, here the benzene breathing mode is clearly distinguishable from the background. From this point on an identification of the analyte on the SERS substrate becomes unambiguous and can be used as a guide for field applications on how long one has to wait before measuring the sample. In this particular case we achieved a signal enhancement of $75\, \%$ in waiting from $15$ to $48\,$ min.

The recorded kinetic (temporal) behavior was fitted using a Hill-Langmuir description (Hill1, which is a modified Hill function with offset)^25^.

$$y=START+(END-START)\frac{{x}^{n}}{{k}^{n}+{x}^{n}}$$

The parameters used are as follows: START = Start area, END = End area, k = Michaelis constant, and n = Cooperative sites, respectively. The following applies for the bounds used: Lower Bounds: $k\,\mathrm{ > \; 0}$ and $n\,\mathrm{ > \; 0}$, Upper bounds: none. This is an equivalent description, as used in enzyme kinetics, for a sigmoidal growth and therefore for the thermodynamic isotherms described by Langmuir or Freundlich. Our findings point, thereby, towards a non-layer-by-layer growth of the analyte at the substrate surface. Thus, most likely a Freundlich thermodynamic description can be used to understand the coverage process.

### Real wastewater samples

After determining the detection limit for the pure BTAH and understanding the coverage dynamics as described in the previous section, we performed a concentration dependent measurement series on a real wastewater sample. This is necessary for an evaluation of the substrates for environmental application use. Wastewater is thereby a complex matrix, which will mask the expected Raman peaks of the BTAH due to the coverage of the SERS-substrates with all kind of different analytes.

The real wastewater measurements were conducted on all SERS-substrates and both equipments. In this case the triazole ring stretching mode at $1387\,c{m}^{-1}$ has been used as reference peak for the determination of the detection limit because it is the more prominent one in the wastewater samples.

**Figure 5:**
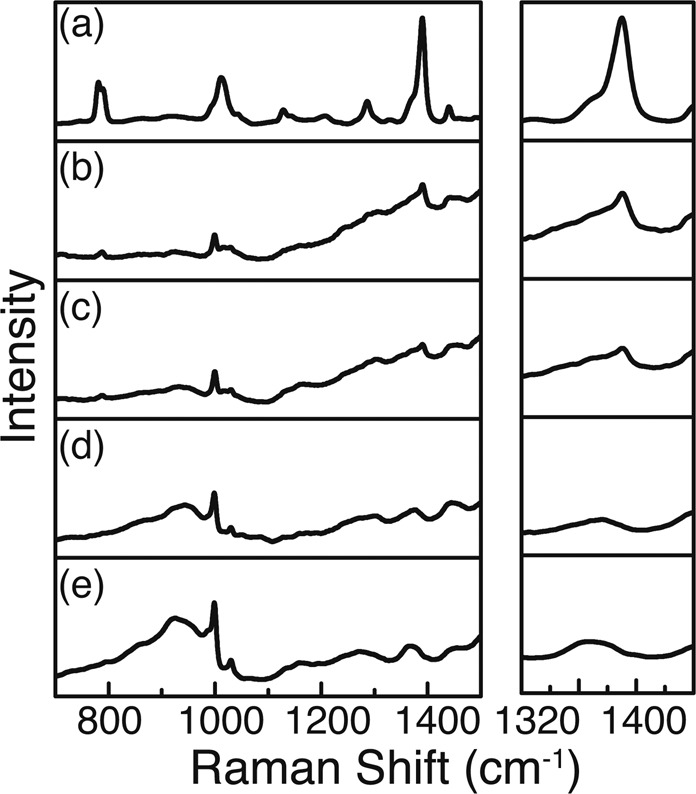
BTAH wastewater spectrum taken with the Kaiser system and the substrate C7. On the right side, the spectrum around the triazole stretching mode is magnified, showing the evolution with increasing concentration. (**a**) Pure BTAH as reference. (**b**–**d**) Decreasing concentration of BTAH from 35.2 over 17.6 to $8.80\,\mu g/l$, respectively. (**e**) Pure C7 substrate.

**Figure 6:**
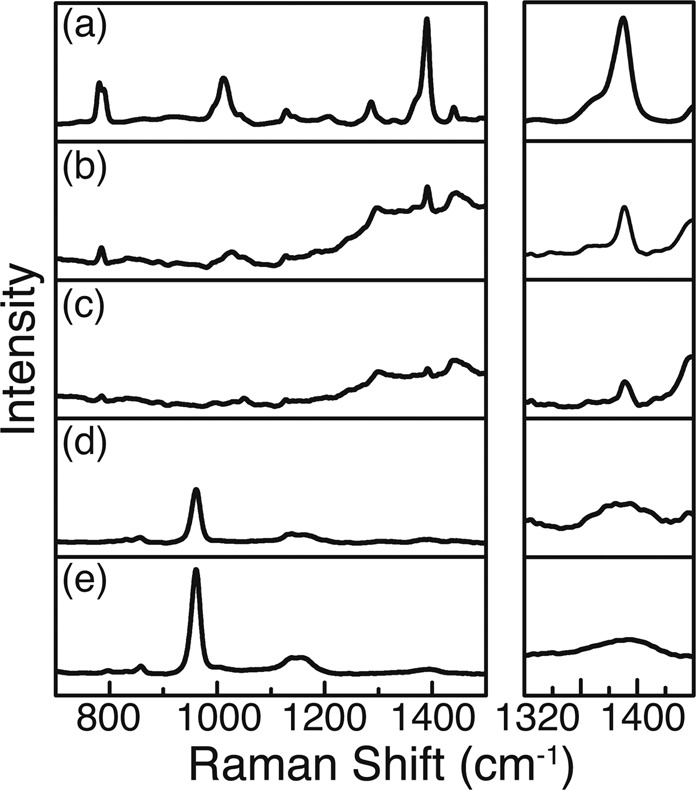
BTAH wastewater spectrum taken with the Kaiser system and the substrate SilAg. On the right side, the spectrum around the triazole stretching mode is magnified, showing the evolution with increasing concentration. (**a**) Pure BTAH as reference. (**b**–**d**) Decreasing concentration of BTAH from 35.2 over 17.6 to $8.80\,\mu g/l$, respectively. (**e**) Pure SilAg substrate.

Here, we show exemplary spectra revealing the detection potential of different samples and also of the portable process Raman instrument (Figs 5 and 6). The additional set of measured spectra is shown in Figs. S1-S4 in the supplementary information. Figures 5 and 6 show, that both tested substrates enable the detection of BTAH directly in the wastewater at a level of $17.6\,\mu g/l$ when concentrating the BTAH in a pre-treatment step. The experiments showed that the portable process Raman spectrometer was also able to detect the BTAH after a pre-concentration step. The detection limits in this case resemble the results of the laboratory equipment.

As can be seen from the recorded Raman spectra, there are differences in the detectability of BTAH on the different SERS-substrates. The results for the different substrates measured with the different spectrometers are summarized in Tables 1 and 2.

**Table 1:** Results of the BTAH wastewater measurements of the different SERS-substrates with the KOSI spectrometer (+: positive analysis, −: negative analysis).

	C7	SilAu	SilAg	MatoS	RandaS
$8.8\,\mu g/l$	−	−	−	−	−
$17.6\,\mu g/l$	+	−	+	−	−
$32.2\,\mu g/l$	+	−	+	−	−

**Table 2:** Results of the BTAH wastewater measurements of the different SERS-substrates with the i-RamanPro spectrometer (+: positive analysis, o: semi-positive analysis, −: negative analysis).

	C7	SilAu	SilAg	MatoS	RandaS
$8.8\,\mu g/l$	−	−	−	−	−
$17.6\,\mu g/l$	−	−	+	−	−
$32.2\,\mu g/l$	o	−	+	−	−

One major finding can be extracted from the experiments. The positive results are all measured with substrates having a periodic nanosized structure (C7 and SilAg). This might be due to the deterministic structure, covering the whole substrate area giving rise to a collective enhancement, since each nanopillar enhances at its tip the same way as the neighboring ones. The RandaS substrates having a stochastic surface seem not to exhibit this effect. Furthermore, it seems that silver gives rise to a stronger enhancement factors compared to gold-surfaces, resulting in detectability of BTAH. Silver is known to be a stronger plasmonic material than gold, so this might be the reason for the better performance. Yet, this raises the question how chips will behave, if materials are adapted. Moreover, with more biopharmaceutical applications to be realized, can Ag-chips keep up with Au-surfaces in terms of biocompatibility or functionalization in the end? Finally, the results on the real wastewater samples emphasize the fact that the nanostructured samples perform superior.

Furthermore, we were interested in the amount of BTAH being detected in the laser spot itself to translate the given results into a more differentiated detection limit. Therefore, we calculated the surface area within the laser spot of $125\,\mu m$ in diameter, resulting in an area of $0.0460\,m{m}^{2}$, acknowledging the pillar structure. We estimated the density ratio of a single BTAH molecule as a rectangle with the parameters as displayed in Fig. 1(c), resulting from the dimensions of the molecule. A monolayer of BTAH within the illuminated area would therefore translate to a mass of $33.3\,pg$ as detection limit. This amount of BTAH, which has been measured both with our sophisticated as well as with commercially available substrates is of comparable magnitude as reported elsewhere, e.g. by Altun *et al*.^20^, showing the possibility to use these SERS-active surfaces in environmental analysis. However, it is shown that BTAH is not limited to form strictly monolayers, see kinetic measurements, leaving the actual value to be possibly larger^1^.

## Conclusion

Benzotriazole (BTAH) is one of the best corrosion inhibitors and commonly used as an antifreeze. Since its problematical environmental effects and decent solubility qualities in water, it represents a threat to the environment and thus also to human health. It is therefore of uttermost importance to have a detection method capable of monitoring the concentration of BTAH at trace level on-site.

Here, we have shown that a sensing method based on surface-enhanced Raman spectroscopy is capable of detecting trace-level concentrations of BTAH in actual wastewater samples. This has been possible with our self-made as well as with commercially available substrates and the use of a portable Raman spectrometer. There has only been a pre-treatment step necessary to achieve a detection limit of $17.6\,\mu g/l$. The conducted experiments demonstrate that it is possible to detect very small amounts of analyte substances other than very small concentrations of that substance in solution. Enabling a direct detection of BTAH at a $\mu g$ and sub-$\mu$ g scale is still a challenging task for current research, yet with our results, the task seems achievable with an equipment already available and a small measuring time (below $48\,$ min) rendering the technique interesting for on-site detection of this pollutant. We have calculated a theoretical detection limit of a monolayer of BTAH in the laser spot at the substrate surface of 33.3 pg, depending on the actual adsorption of BTAH. Furthermore, we have carefully studied the concentration behavior and modelled the concentration dependency and time-dependent coverage, showing that it can be described by Freundlich isotherms.

## Supplementary information

Supplementary Information.
