# Supplementary material for: Evaluation of an on-site surface enhanced Raman scattering sensor for benzotriazole
Source: Sci Rep. 2020 May 19;10:8260. doi: 10.1038/s41598-020-65181-z (PMC7237660; doi:10.1038/s41598-020-65181-z)
Supplement: Supplementary file 1 — Supplementary Information. [file 41598_2020_65181_MOESM1_ESM.pdf]

Supporting Information: Evaluation of an on-site surface enhanced Raman scattering sensor for benzotriazole

Florian Wieduwilt[1,*], Christoph Lenth[1], Georgios Ctistis[1,*], Ulrich Plachetka[2], Michael Möller[2], and Hainer Wackerbarth[1]

[1]Laser-Laboratorium Göttingen e.V., Hans-Adolf-Krebs-Weg 1, 37077 Göttingen, Germany
[2]AMO GmbH, Otto-Blumenthal-Straße 25, 52074 Aachen, Germany
[*]florian.wieduwilt@llg-ev.de, georgios.ctistis@llg-ev.de

ABSTRACT

This is the supplementary information to the manuscript Evaluation of an on-site surface enhanced Raman scattering sensor for benzotriazole. The following figures complete thereby the described SERS data sets discussed in the paper.

Figures S1 - S4

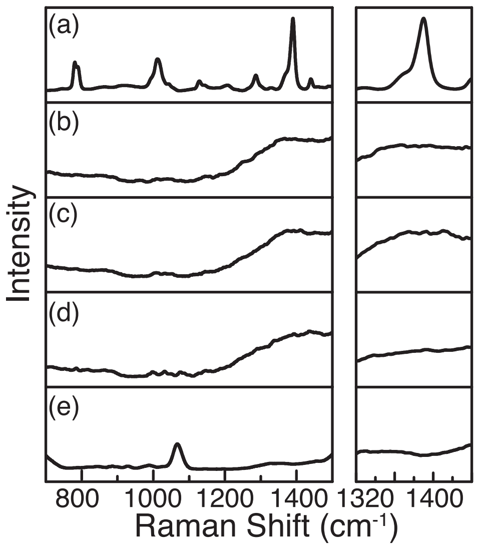

Figure S1. BTAH waste-water spectrum taken with the Kaiser spectrometer and the substrate RandaS. On the right side, the spectrum around the triazole stretching mode is magnified, showing the evolution with increasing concentration. (a) Pure BTAH as reference. (b) – (d) Decreasing concentration of BTAH from 35.2 over 17.6 to 8.80 μg/l, respectively. (e) Pure RandaS substrate.

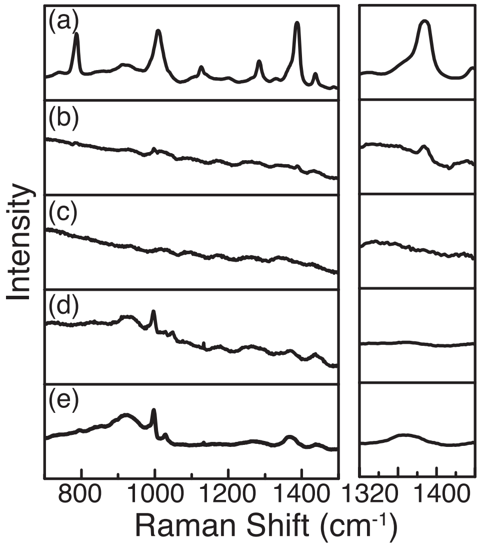

Figure S2. BTAH waste-water spectrum taken with the i-RamanPro spectrometer and the substrate C7. On the right side, the spectrum around the triazole stretching mode is magnified, showing the evolution with increasing concentration. (a) Pure BTAH as reference. (b) – (d) Decreasing concentration of BTAH from 35.2 over 17.6 to 8.80 μg/l, respectively. (e) Pure C7 substrate.

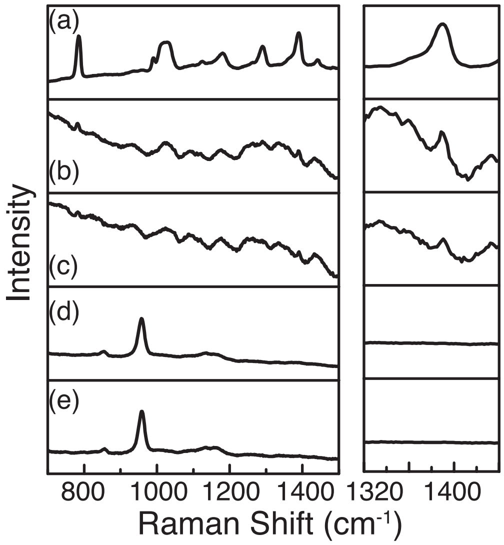

Figure S3. BTAH waste-water spectrum taken with the i-RamanPro spectrometer and the substrate SilAg. On the right side, the spectrum around the triazole stretching mode is magnified, showing the evolution with increasing concentration. (a) Pure BTAH as reference. (b) – (d) Decreasing concentration of BTAH from 35.2 over 17.6 to 8.80 μg/l, respectively. (e) Pure SilAg substrate

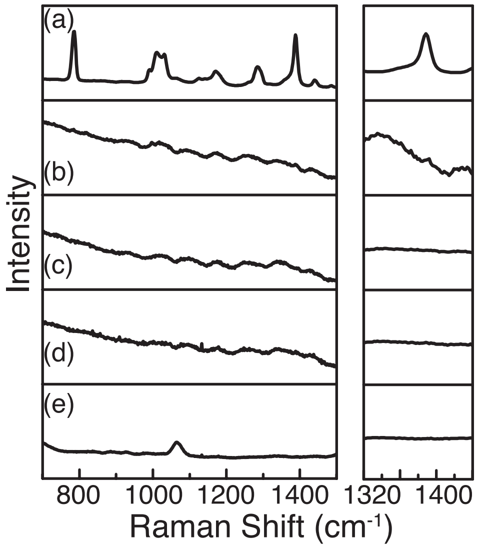

Figure S4. BTAH waste-water spectrum taken with the i-RamanPro spectrometer and the substrate RandaS. On the right side, the spectrum around the triazole stretching mode is magnified, showing the evolution with increasing concentration. (a) Pure BTAH as reference. (b) – (d) Decreasing concentration of BTAH from 35.2 over 17.6 to 8.80 μg/l, respectively. (e) Pure RandaS substrate
